# Oxidative Damage Is Influenced by Diet But Unaffected by Selection for Early Age of Oviposition in the Marula Fly, *Ceratitis cosyra* (Diptera: Tephritidae)

**DOI:** 10.3389/fphys.2022.794979

**Published:** 2022-02-28

**Authors:** Kevin Malod, Esther E. du Rand, C. Ruth Archer, Susan W. Nicolson, Christopher W. Weldon

**Affiliations:** ^1^Department of Zoology and Entomology, University of Pretoria, Hatfield, South Africa; ^2^Institute for Evolutionary Ecology and Conservation Genomics, University of Ulm, Ulm, Germany

**Keywords:** trade-off, oxidative damage, antioxidants, nutrition, Tephritidae

## Abstract

The expression of life-history traits, such as lifespan or reproductive effort, is tightly correlated with the amount and blend of macronutrients that individuals consume. In a range of herbivorous insects, consuming high protein to carbohydrate ratios (P:C) decreases lifespan but increases female fecundity. In other words, females face a resource-based trade-off between lifespan and fecundity. Redox metabolism may help mediate this trade-off, if oxidative damage is elevated by reproductive investment and if this damage, in turn, reduces lifespan. Here, we test how diets varying in P:C ratio affect oxidative damage and antioxidant protection in female and male of the marula fly, *Ceratitis cosyra* (Diptera: Tephritidae). We use replicated lines that have been subjected to experimental evolution and differ in their lifespan and reproductive scheduling. We predicted that high fecundity would be associated with high oxidative damage and reduced antioxidant defences, while longer lived flies would show reduced damage and elevated antioxidant defences. However, higher levels of oxidative damage were observed in long-lived control lines than selection lines, but only when fed the diet promoting lifespan. Flies fed diets promoting female fecundity (1:4 and 1:2 P:C) suffered greater oxidative damage to lipids than flies fed the best diet (0:1 P:C) for lifespan. Total antioxidant capacity was not affected by the selection regime or nutrition. Our results reiterate the importance of nutrition in affecting life-history traits, but suggest that in *C. cosyra*, reactive oxygen species play a minimal role in mediating dietary trade-offs between lifespan and reproduction.

## Introduction

Life-history theory considers how organisms schedule investment in key fitness traits, such as when and how large to grow, when to invest in offspring and when to invest in somatic maintenance (Stearns, 2000). Trade-offs between life-history traits are common, for example, an increase in current reproductive effort often leads to reduced future reproductive investment or lifespan (Edward and Chapman, 2011). While trade-offs involving current and future reproduction or survival are widespread, the mechanisms driving these live-history trade-offs are often unclear (Zera and Harshman, 2001; Harshman and Zera, 2007).

Physiological trade-offs (i.e., those evident at individual level) are often tightly linked with nutrition. In particular, life-history trait expression is frequently influenced by both the amount and blend of ingested macronutrients (Raubenheimer and Simpson, 2018). In herbivorous insects, for example, a low protein, high carbohydrate ratio (P:C) typically improves lifespan (Lee et al., 2008; Dussutour and Simpson, 2009; Fanson et al., 2009; Archer et al., 2014; Paoli et al., 2014; Jensen et al., 2015; Malod et al., 2017; Rapkin et al., 2017; Jang and Lee, 2018; Krabbe et al., 2019; Moatt et al., 2019; Duffield et al., 2020). However, this nutrient ratio often reduces female fecundity, which tends to be greater in females fed diets richer in protein (Maklakov et al., 2008; Jensen et al., 2015; Le Couteur et al., 2016; Rapkin et al., 2017). This likely reflects that females require protein for egg production, and in many insect species, protein helps stimulate oogenesis (Wheeler, 1996). Because females cannot eat a single nutrient blend to maximise the expression of both lifespan and fecundity at the same time, they face a diet-mediated trade-off. In contrast, in the males studied to date the nutrients blends that improve lifespan also promote reproductive effort (Maklakov et al., 2008; South et al., 2011; Jensen et al., 2015; Rapkin et al., 2017), although there are exceptions (Harrison et al., 2014; Bunning et al., 2015). Because males can consume a single nutrient blend to promote lifespan and fertility, males experience a less pronounced trade-off between these traits than females. This aligns with the general observation that diet-mediated trade-offs between lifespan and reproduction are more pronounced in females than males (Nakagawa et al., 2012; Adler et al., 2013). Although apparent sex differences in responses to dietary manipulation may reflect the lack of data testing how nutrition shapes male reproductive success rather than a genuine biological signal (Moatt et al., 2016).

Even though we know that individuals fed high P:C ratios often die younger, the physiological mechanisms behind lifespan reduction are yet to be elucidated (Moatt et al., 2020). More generally, the mechanistic basis of trade-offs between survival and reproductive investment are also unclear. Diet-mediated trade-offs between lifespan and reproduction could simply reflect a conflict between allocation of resources to reproductive effort at the expense of somatic maintenance, or vice versa (Kirkwood, 2017). For example, in female insects where proteins are needed for egg production, protein allocation to oogenesis could take away resources that could have been used to maintain tissue integrity (Le Couteur et al., 2016). Another potential explanation for this trade-off is that, while proteins are needed in large quantities for reproduction, they can also be toxic to the organism, and this toxicity (or metabolic cost associated with prevention of toxicity) reduces lifespan (Fanson et al., 2012; Anderson et al., 2020). This toxicity could originate from nitrogenous compounds resulting from protein metabolism and/or from increased reactive oxygen species (ROS) production associated with high protein consumption (Ayala et al., 2007; Moatt et al., 2020).

Oxidation–reduction reactions taking place in mitochondria to produce energy also release ROS, which are highly unstable by-products from the oxygen molecules used in the process. Because of their reactive nature, ROS can oxidise cellular components such as proteins, lipids or nucleic acids (Harman, 1956). To mitigate these potentially harmful effects, cells are equipped with antioxidant defences (enzymatic and non-enzymatic molecules) that render ROS inert or repair oxidative damage. However, if defences are exceeded to such an extent that redox homeostasis is disrupted, damage may occur (Selman et al., 2012). It has been hypothesised that trade-offs between life-history traits may be mediated by ROS production and more particularly by the balance between ROS and antioxidant protection (Dowling and Simmons, 2009; Monaghan et al., 2009; Speakman et al., 2015). ROS could have different ways of influencing trade-offs, which may vary across the life of an organism. For example, there could be a short-term trade-off if resources allocated to oxidative damage prevention and repair could have been used for other purposes (e.g., immunity and sexual signalling). However, there might also be a long-term trade-off with an indirect cost if investment in fitness traits (e.g., reproduction) elevates ROS production without parallel increase in antioxidant protection (Monaghan et al., 2009). If ROS production mediates trade-offs between life-history traits, such as lifespan and fecundity, we expect that diets that affect these traits, also affect ROS—antioxidant protection equilibrium (Isaksson et al., 2011). In other words, shortened lifespan resulting from dietary manipulation should be accompanied by elevated oxidative damage.

Some data support the idea that dietary manipulations affect lifespan or reproduction by modulating oxidative damage and/or antioxidant defences. For example, in *Rattus norvegicus* where protein restricted diets increase lifespan, ROS production in the liver declines when protein intake decreases (Sanz et al., 2004). In *Drosophila melanogaster*, where high protein intake reduces lifespan, lipid peroxidation and protein oxidation can be mitigated by high carbohydrate intake (Rovenko et al., 2015). In contrast, in adult oriental fruit flies, *Bactrocera dorsalis*, oxidative damage to lipids decreased as the yeast to sugar ratio in the diet increased (Chen et al., 2013). Diets that reduced lifespan also improved antioxidant protection, suggesting that the increase in antioxidant defences was not sufficient to prevent lifespan reduction (Chen et al., 2013). Although, this result could also reflect that antioxidant protection was elevated in response to an increase in other types of damage that were not considered (e.g., protein oxidation). A similar trend was observed in the Australian field cricket, *Teleogryllus commodus*, where protein oxidative damage was higher in individuals fed low P:C ratios that improved lifespan (Archer et al., 2015). However, antioxidant protection also decreased as the P:C ratio increased (Archer et al., 2015). Collectively, these studies show that dietary macronutrient ratio can affect oxidative damage and antioxidant protection. However, the magnitude and direction of effects vary between species. Moreover, our understanding is limited by the few species investigated, as well as differences in the dietary manipulations used.

Lastly, nutrient storage has been linked with survival during stress and reproductive performance (Jacome et al., 1995; Warburg and Yuval, 1997; Yuval et al., 1998; Nestel et al., 2016; Weldon et al., 2016). This is particularly true for lipids, because lipid reserves have been associated with longer lifespan (Hansen et al., 2013), and lipid storage responds to dietary manipulations (Lee and Jang, 2014). However, carbohydrate-rich diets can lead to excessive fat (i.e., lipid) accumulation, and this can reduce lifespan (see supplemental data in Maklakov et al., 2008). Therefore, testing how nutrient storage varies as a function of life-history strategies and dietary manipulation may offer a means for extension of lifespan that complements ROS-antioxidant equilibrium.

Here, we tested the hypothesis that oxidative stress mediates the trade-off between lifespan and reproduction. We fed females and males of the marula fruit fly (*Ceratitis cosyra*) diets that promote lifespan or female reproductive effort. We predicted that flies fed diets that have a positive impact on reproduction but a negative effect on lifespan would reduce antioxidant protection and/or increase ROS production, elevating oxidative damage. Furthermore, we employed selected lines that differ in their reproductive scheduling and lifespan (Malod et al., 2021). We predicted that flies from lines selected downwards on age of female oviposition would exhibit higher levels of oxidative damage and/or decreased antioxidant protection due to their shorter lives and earlier reproductive effort. Finally, we predicted stored, soluble protein content of flies to be higher when fed a protein rich diet. Lipid reserves were predicted to be lower when flies were fed a protein rich diet (Lee and Jang, 2014), and in downward-selected flies, as high lipid reserves are associated with longer lives.

## Materials and Methods

### Fly Husbandry

Infested mangoes from across Mpumalanga province, South Africa, were collected and *C. cosyra* pupae retrieved. The wild flies emerging from these pupae were used to establish a culture that was then maintained at ~23°C in a climate room with a 14:10 light:dark photocycle. To create optimal mating conditions, the first and last hour of the light phase simulated dawn and dusk with 8 W fluorescent tubes (T4, Eurolux, Sandton, South Africa) that were placed obliquely to the fly culture and turned on before, and turned off after, the main room lights. The remaining room lights, comprising a combination of 20 W (G5, Eurolux, Sandton, South Africa) and 58 W (58 W/840, Osram, Germany) fluorescent tubes were also turned on for the remainder of the light period. Adults were kept in groups of *ca.* 200 flies in 5 L plastic cages with unrestricted access to food (hydrolysed yeast and sugar in separate dishes) and water (water-soaked cotton wool). At 15 days after emergence, wild males were crossed with females from a laboratory culture provided by Citrus Research International (Nelspruit, South Africa). This step introduced wild genetic diversity to our stock population, while retaining the tendency for culture females to oviposit into an artificial substrate. The next generation was obtained by allowing laboratory females mated with wild males to lay eggs on a 125 ml plastic container (Plastilon, South Africa) covered with a layer of laboratory film (Parafilm M, Bemis, United States) pierced several times with a pin. Tissue paper soaked with 3 ml of guava juice concentrate (Hall’s, Tiger Consumer Brands Limited, Bryanston, South Africa) was placed in the plastic container to encourage females to oviposit through the film. Eggs were then washed out of the artificial substrate with water and placed on 125 ml of a carrot-based larval rearing medium (Citrus Research International, Nelspruit, South Africa) in a plastic container at an approximate density of 2.5 eggs/ml of medium. The container of larval rearing medium was then placed in a 2 L plastic box with a layer of sand and a ventilated lid. After 15 days, during the pupal phase, the sand was sifted and the retrieved pupae placed in a Petri dish (ø 65 mm) and transferred into a 5 L cage with unrestricted access to food and water for emerging adults.

### Selection Regime

Selection began three generations after laboratory females had been crossed with wild males and a strong culture had been established. We selected on the age of oviposition (procedure similar to *D. melanogaster*, see Rose, 1984; Arking, 1987) by only providing an oviposition substrate (a 125 ml plastic container with guava juice-soaked tissue paper) when flies were 5 days old (downward-selected, DS) or 15 days old (control, CT). In our laboratory, 15 days is the average age when eggs are collected from this species and is also when oviposition peaked in earlier studies (Manrakhan and Lux, 2006; Roets et al., 2018). Downward selection was performed at 5 days old and not earlier because mating activity during the first week after emergence is generally low (<5%; Manrakhan and Lux, 2006). For each selection regime (DS and CT) we established four replicate populations. We maintained the selection regime under identical conditions until the generation at which the flies were assayed. Because flies were selected on age of oviposition, selection lines inevitably reached generation 44 at a different date. By 20 generations of selection, DS lines had earlier peaks in fecundity and reduced lifespan relative to CT lines (Malod et al., 2021). For detailed information on the selection procedure, see Malod et al. (2021).

### Experimental Diets and Feeding Procedure

We selected three experimental diets that varied in their P:C ratios (0:1; 1:4; 1:2) while total nutrient concentrations remained the same (i.e., P + C = 360 g/L). The effects of these P:C ratios on phenotype were characterised on a non-selected stock population (see Malod et al., 2017). The 0:1 P:C ratio increased lifespan but reduced female reproductive effort, whereas the 1:2 P:C ratio promoted daily female fecundity but reduced lifespan (Malod et al., 2017). The 1:4 P:C ratio is an intermediate between the best diet for lifespan (0:1 P:C) in both sexes and the best diet for female daily fecundity (1:2 P:C). This nutrient blend also represents the closest ratio to the regulated intake point (nutrient blend that an individual self-selects when provided with a choice of different nutritionally imbalanced diets; Malod et al., 2017). Of the three diets provided to the flies, the 1:4 P:C is the best to promote at the same time lifetime egg production and daily egg production in females (Supplementary Figures S1A,B). All diets were prepared by mixing 18 amino acids (source of protein) and sucrose (source of carbohydrates) in distilled water (Supplementary Table S1).

Each diet was provided to five females and five males taken at random from each replicate of the selected lines (Five flies × two sexes × three diets × two selection regimes × four replicate lines = 240 individuals). Diets were also presented to a group of “wild flies” (30 individuals) as a control for laboratory adaptation. The wild group comprised the stock population, assayed the first generation after crossing wild-caught males with laboratory females and was therefore unselected. After emergence, flies were individually placed in a plastic container (125 ml) and given access to 200 μl of distilled water and 80 μl of one of the three diets, both water and diet being supplied in 200 μl pipette tips. Diet and water were replaced every 5 days or earlier if depleted. After 25 days, flies were chilled at −20°C for two to 3 min and then placed in individual, labelled 2 ml microcentrifuge tubes before being stored in a −80°C freezer. Flies were killed and preserved at 25 days of age to minimise selective disappearance, where low-quality individuals are lost from the cohort at an early age and therefore not included in tests of traits associated with lifespan. This limit was set because when non-selected flies are fed the diet associated with poor survival (1:2 P:C ratio), 50% of them are dead after 70 days (Malod et al., 2017).

### Oxidative Damage, Antioxidant and Body Composition Assays

To measure oxidative damage to proteins and lipids and total antioxidant capacity (TAC), we used commercial kits (Sigma Aldrich, Saint-Louis, MO, United States) and adapted the manufacturer recommendations. To measure body composition (soluble proteins, lipids), we used the methods described by Foray et al. (2012) that have been adapted for tephritids (Weldon et al., 2019). By doing so, we were able to measure all biochemical parameters in a single fly. All assays were colorimetric, where determination of the component of interest was performed by reading absorbance of samples and comparing it to a standard curve with a microplate reader (Eon Microplate Spectrophotometer, BioTek Instruments, Winooski, VT, United States). Twelve microplates were necessary to determine the different biochemical parameter for the 270 flies. Flies from each combination of selected line, sex and diet were arbitrarily assigned to each microplate to equally spread among the groups the potential variation between plates.

Flies were removed from the −80°C freezer, weighed to determine wet mass and individually placed in a 2 ml screwcap tube (SSIbio, Lodi, CA, United States) with a 3 Ø zirconium bead (Benchmark Scientific Inc., Edison, NJ, United States) and 200 μl of 5 mM phosphate-buffered saline (PBS; 1.35 mM potassium chloride, 68.5 mM sodium chloride, pH 7.4; P4417, Sigma Aldrich, Saint-Louis, MO, United States). Flies were then homogenised using a benchtop homogeniser (BeadBug Microtube Homogenizer, Benchmark Scientific Inc., Edison, NJ, United States) for 1 min. After homogenisation, samples were centrifuged at 200 RCF for 15 min at room temperature (~20°C). After centrifugation, 100 μl of the homogenised solution was transferred to a new 2 ml microcentrifuge tube (Eppendorf, Hamburg, Germany) for later determination of oxidative damage to protein. To the remaining homogenate, 500 μl of PBS was added for later determination of lipid peroxidation, total antioxidant capacity and lipid content.

Protein content was determined using a bicinchoninic acid protein kit (B9643). The 600 μl homogenised solution was centrifuged for 5 min at 3,000 RCF and 20 μl of the supernatant transferred to a new 1.5 ml microtube (Kartell, Noviglio, Italy). To have a two times dilution, 20 μl of PBS was added to the 20 μl of homogenised solution. Then, 200 μl of BCA working reagent (bicinchoninic acid solution with copper (II) sulphate pentahydrate, 4% solution) was added to the 40 μl of diluted sample. Samples were then shaken for 30 s and incubated for 30 min at 37°C. After incubation, sample absorbance was read at 562 nm and compared with the absorbance of bovine serum albumin (BSA) standards (0, 25, 125, 250, 500, 750 and 1,000 μg/ml).

Protein oxidation was measured by determining protein carbonyl group content with a kit (Sigma Aldrich, MAK094). To the 100 μl previously set aside, 10 μl of 10% streptozocin was added and samples incubated for 15 min at room temperature. They were then centrifuged at 13,000 RCF for 5 min and the supernatant from each tube was transferred to new 1.5 ml microtube. To the supernatant, 100 μl of 2,4-dinitrophenylhydrazine solution was added and samples incubated for 10 min at room temperature. After incubation, 30 μl of 100% trichloroacetic acid solution was added and samples were then placed on ice and incubated for 5 min. Following incubation, samples were centrifuged for 5 min at 13,000 RCF and the supernatant was then discarded. To the remaining pellet, 500 μl of ice cold 100% acetone was added and samples were placed for 30 s in an ultrasonic bath (40 Hz; DU-32, Argo Lab, Italy) at room temperature. Then, samples were incubated at −20°C for 5 min. After incubation, samples were centrifuged at 13,000 RCF for 2 min and the acetone was removed. All steps from adding 500 μl of acetone to removing it were repeated. After discarding the second acetone wash, 210 μl of 6 M guanidine solution was added to the pellet and samples placed in the ultrasonic bath for 30 s. Absorbance of samples was read at 375 nm and compared to the absorbance of ultra-pure (double distilled and deionised) water. Oxidative damage to protein was expressed in nmole of protein carbonyl per fly.

The total antioxidant capacity (TAC) assay kit (Sigma Aldrich, MAK187) determines the total antioxidant capacity (enzymatic and non-enzymatic). To determine TAC, we placed 10 μl of fly homogenate directly into a well of a 96-well microplate and added 90 μl of PBS to have a 10 times dilution. Then 100 μl of Cu^2+^ working solution was added to each sample. To protect from evaporation and light, the microplate was covered with a film and a piece of aluminium foil and then incubated at room temperature for 90 min. After incubation, the film and foil were removed. Sample absorbance was read at 570 nm and compared to 6-hydroxy-2,5,7,8-tetramethylchroman-2-carboxylic acid (trolox; a water-soluble vitamin E analog) standards (0, 20, 40, 60, 80 and 100 nmole/μl). Total antioxidant capacity in a fly was expressed in nmole of trolox equivalent per milligram of fly.

Oxidative damage to lipid was assessed by determination of lipid peroxidation through measurement of malondialdehyde (MDA) formation (Sigma Aldrich, MAK085 kit). From the fly homogenate, 200 μl was pipetted and placed in a new 2 ml microtube. Then 3 μl of BHT and 150 μl of MDA lysis buffer solution as well as 153 μl of 2 N perchloric acid were added to the 200 μl solution. Samples were then centrifuged at 13,000 RCF for 10 min. A volume of 200 μl from the supernatant was then transferred to a new 2 ml microtube and 600 μl of thiobarbituric acid (TBA) solution was added. Samples were then incubated for 60 min at 90°C and cooled on ice for 10 min. Sample absorbance was read at 532 nm and compared against MDA standards (0, 0.5, 1, 2, 4 and 6 nmole). Oxidative damage to lipids should be expressed in nmole of MDA per mg of lipids. However, because lipid content was too low to be detected in some samples, we expressed the oxidative damage to lipids in nmole of MDA per fly.

Prior to lipid determination assay, 180 μl of fly homogenate was transferred to a new 2 ml microtube. Then, 20 μl of 20% Na_2_SO_4_ and 1.5 ml of chloroform:methanol (1:2 v/v) were added to the samples and vigorously combined using a vortex mixer (MX-S vortex, DLAB Instruments Ltd., CA, United States). After being vortexed, samples were centrifuged at room temperature for 15 min at 200 RCF. For lipid determination we transferred in duplicate 100 μl of supernatant to a new microtube. They were then evaporated under a fume hood for 24 h. After evaporation, 10 μl of 98% sulphuric acid was added and samples incubated at 90°C for 2 min. After incubation, samples were cooled on ice for 10 min. Then, 210 μl of vanillin reagent (1.2 g/L in 68% orthophosphoric acid) was added to the samples. After addition of the reagent, samples were shaken for 15 min at room temperature and absorbance read at 525 nm. Absorbance of samples was compared with glycerol trioleate standards (0, 0.1, 0.2, 0.5 and 1 mg/ml).

### Statistical Analyses

All statistical analyses were performed in R (v 4.1.1, The R Foundation for Statistical Computing). Generalised linear mixed effects models were used for all traits, with gamma (protein oxidation, lipid peroxidation, protein and lipid content) or Gaussian (TAC and body weight) families. Because the gamma family cannot be used with zero values, if the lipid peroxidation (*n* = 130) or lipid (*n* = 87) content concentrations were at zero, these values were replaced with the smallest value of the dataset for the respective trait and divided by 10. Models were built using the “glmer” or “lmer” functions from the “lme4” package (Bates et al., 2015). Selection, diet, sex and their interactions were included as fixed factors in all models. Replicate line ID nested within selection regime was included as a hierarchical random effect term, except for TAC where replicate alone was included as a random factor because the variance of replicate nested within selection was null. For oxidative damage to proteins and lipids, protein and lipid content were included in the model as covariates. Body weight was included as a covariate for the analyses of protein and lipid content. Models were reduced to the minimal adequate model using backwards model simplification. Analysis of deviance tables was generated using type III sums of squares to summarise the effect of each factor in the minimal adequate model. If a significant main effect or interaction was detected, *post hoc* pairwise comparison tests of the estimated marginal means were performed using the function “emmeans” from the package of the same name (Russel, 2019). The pairwise comparisons from the “emmeans” function return an estimate that is the difference (or the ratio) between the two compared groups and indicates the direction of the difference.

## Results

### Oxidative Damage and Total Antioxidant Capacity

**Figure 1:**
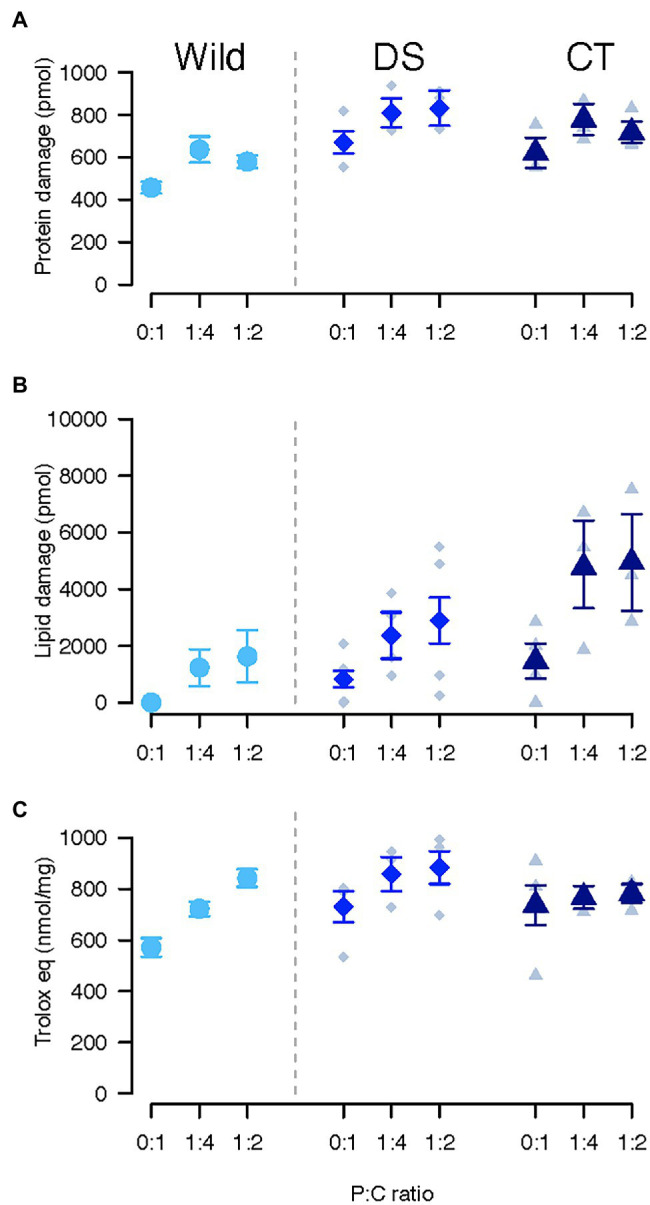
Oxidative damage to proteins **(A)**, lipids **(B)**, and total antioxidant capacity **(C)** in *Ceratitis cosyra* from control (CT), wild (Wild) or lines selected downwards (DS) on age of oviposition and fed different diets varying in their protein to carbohydrate ratio. Error bars indicate the SE of the mean for wild flies, and the SE of the grand mean for replicate in CT and DS lines.

Protein oxidation was only influenced by selection regime (Table 1). Wild flies experienced the lowest oxidative damage to proteins (CT/Wild: ratio = 1.24, *p* = 0.017; Wild/DS: ratio = 0.73, *p* < 0.001), while there was no difference between CT and DS flies (CT/DS: ratio = 0.91, *p* = 0.119; Figure 1A).

**Table 1:** Effects of selection regime, diet and sex in *Ceratitis cosyra* selected on age of oviposition and fed either a 0:1, 1:4 or 1:2 P:C diets.

	*χ* ^2^	df	*p*
**Protein oxidation**			
Selection	8.64	2	**0.013**
Diet	3.65	2	0.161
Sex	1.29	1	0.255
Protein content	2.09	1	0.147
Selection × Diet	1.81	4	0.769
Selection × Sex	0.69	2	0.704
Diet × Sex	0.41	2	0.815
Selection × Diet × Sex	1.85	4	0.763
Variance random effect	0.001		
**Lipid peroxidation**			
Selection	18.37	2	**<0.001**
Diet	4.91	2	0.085
Sex	1.52	1	0.217
Lipid content	6.43	1	**0.011**
Selection × Diet	12.21	4	**0.016**
Selection × Sex	0.34	2	0.844
Diet × Sex	0.57	2	0.752
Selection × Diet × Sex	2.11	4	0.716
Variance random effect	1.793		
**TAC**			
Selection	1.38	2	0.501
Diet	0.81	2	0.665
Sex	1.12	1	0.291
Selection × Diet	3.04	4	0.552
Selection × Sex	0.26	2	0.877
Diet × Sex	0.127	2	0.938
Selection × Diet × Sex	0.595	4	0.964
Variance random effect	8,714		
**Protein content**			
Selection	1.48	2	0.477
Diet	15.17	2	**<0.001**
Sex	1.27	1	0.258
Body weight	47.08	1	**<0.001**
Selection × Diet	3.09	4	0.543
Variance random effect	0.001		
**Lipid content**			
Selection	25.22	2	**<0.001**
Diet	0.27	2	0.871
Sex	0.69	1	0.404
Body weight	1.03	1	0.311
Selection × Diet	10.82	4	**0.029**
Selection × Sex	0.08	2	0.959
Diet × Sex	0.28	2	0.871
Selection × Diet × Sex	0.65	4	0.957
Variance random effect	0.162		
**Body weight**			
Selection	9.49	2	**0.008**
Diet	68.96	2	**<0.001**
Sex	38.58	1	**<0.001**
Selection × Diet	8.15	4	0.086
Selection × Sex	3.02	2	0.221
Diet × Sex	21.89	2	**<0.001**
Selection × Diet × Sex	5.38	4	0.251
Variance random effect	0.067		

Data were analysed using generalised linear mixed effects models. Only the traits retained in the minimal adequate models are displayed. Replicate lines were added as a random effect nested within selection regime. P-values in bold type are statistically significant

An interaction between selection regime and diet affected lipid peroxidation (Table 1). Selection regimes only differed when flies were fed the diet promoting lifespan (0:1 P:C). Wild flies had the lowest levels of lipid peroxidation (CT/Wild: ratio = 222.59, *p* < 0.001; Wild/DS: ratio = 0.03, *p* = 0.003) and CT flies the highest (CT/DS: ratio = 6.54, *p* = 0.026; Figure 1B). In all lines, flies fed the diet promoting lifespan suffered less oxidative damage than flies fed the other diets (**Wild**: 0:1/1:4: ratio = 0.01, *p* < 0.001; 0:1/1:2: ratio = 0.005, *p* < 0.001; **CT**: 0:1/1:4: ratio = 0.32, *p* = 0.018; 0:1/1:2: ratio = 0.32, *p* = 0.019; **DS**: 0:1/1:4: ratio = 0.09, *p* = 0.018; 0:1/1:2: ratio = 0.15, *p* < 0.001). There was no significant difference between 1:4 and 1:2 P:C. Lipid peroxidation significantly decreased as lipid storage increased (estimate = −0.47, *p* = 0.011).

None of the factors significantly affected total antioxidant capacity (Table 1; Figure 1C).

### Body Composition

**Figure 2:**
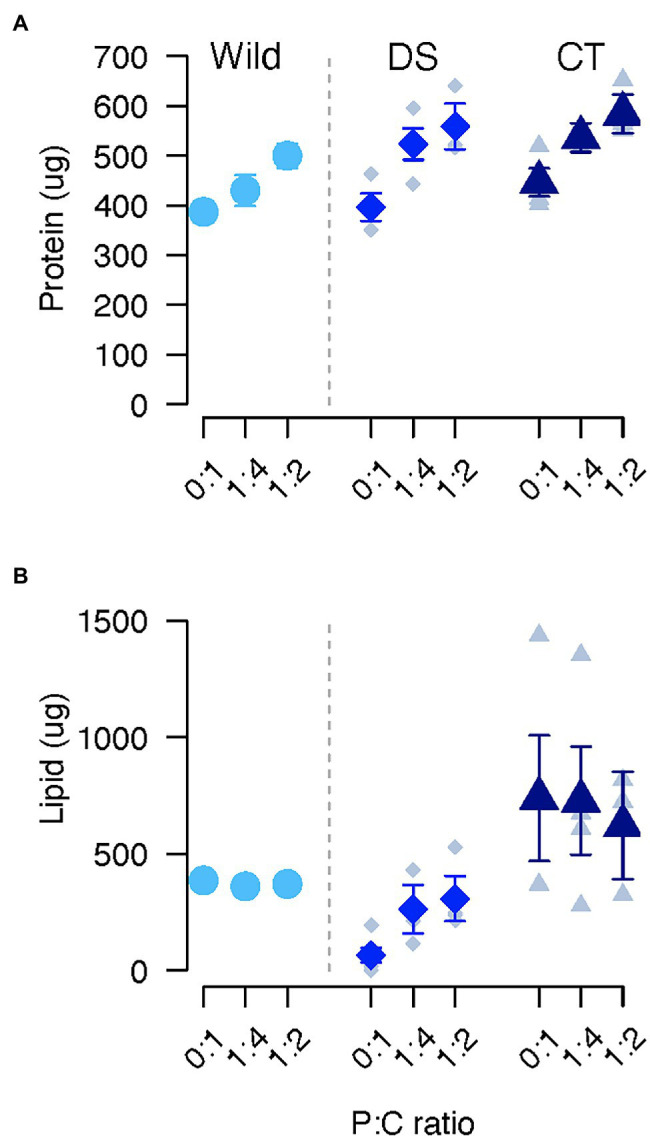
Protein **(A)** and lipid **(B)** in *C. cosyra* from control (CT), wild (Wild) or lines selected downwards (DS) on age of oviposition and fed different diets varying in their protein to carbohydrate ratio. Error bars indicate the SE of the mean for wild flies, and the SE of the grand mean for replicate in CT and DS lines.

Experimental diet significantly affected protein content (Table 1). Flies fed the diet promoting lifespan (0:1 P:C) had lower protein stores than flies fed the intermediate diet (0:1/1:4: ratio = 0.91, *p* = 0.008) or the best diet for daily egg production (0:1/1:2: ratio = 0.83, *p* < 0.001; Figure 2A). Flies fed the best diet for daily egg production had the highest protein storage (1:4/1:2: ratio = 0.92, *p* = 0.021). Protein content slightly increased with body weight (estimate = 0.13, *p* < 0.001; Table 1).

Selection regime and diet together influenced lipid storage (Table 1). *Post hoc* analyses revealed that lipid content of DS flies was lower than CT (CT/DS: ratio = 14.4, *p* < 0.001) or wild (Wild/DS: ratio = 7.57, *p* = 0.004) flies when fed a 0:1 P:C diet (Figure 2B). When flies were fed the 1:4 diet, CT flies also stored more lipids than DS flies (CT/DS: ratio = 2.84, *p* = 0.024). No significant differences were detected between other groups or when flies were fed the 1:2 P:C diet. Within the same selection regime, lipid storage did not vary across the different diets except for DS flies (Figure 2B). Lipid storage was lowest in DS flies when fed with the 0:1 P:C diet (0:1/1:4: ratio = 0.178, *p* = 0.073; 0:1/1:2: ratio = 0.141, *p* < 0.001), and no significant difference was detected between flies fed the 1:4 and 1:2 P:C diets (Figure 2B).

### Body Weight

**Figure 3:**
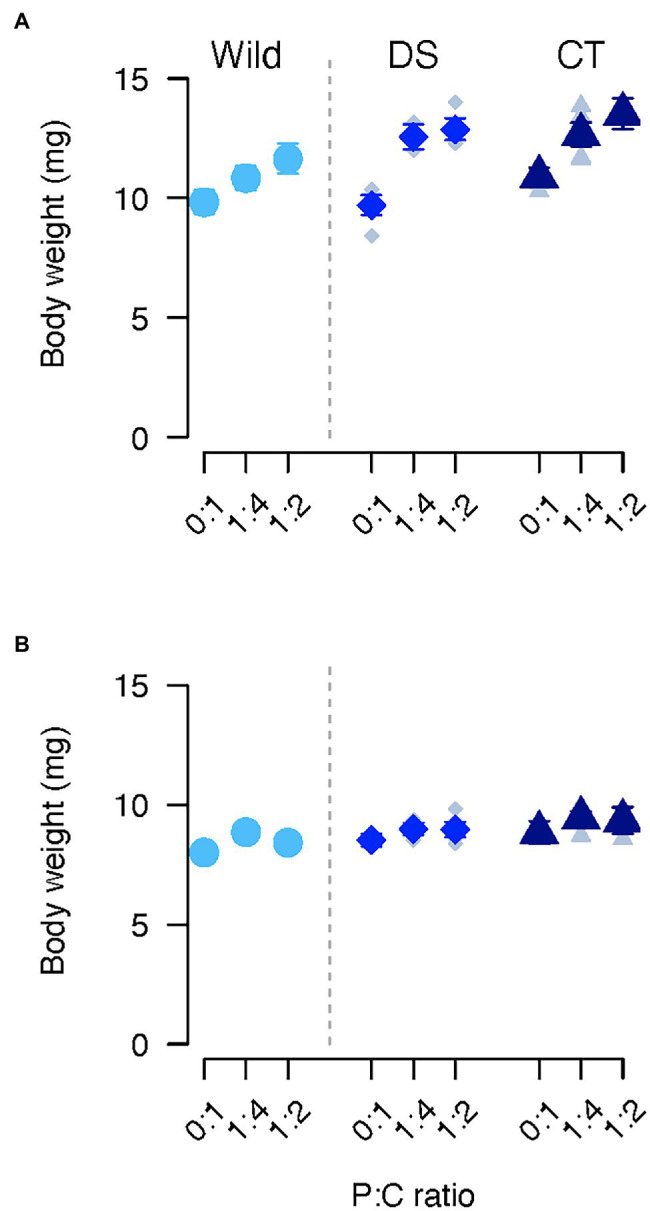
Body weight of females **(A)** and males **(B)** of *C. cosyra* from control (CT), wild (Wild) or lines selected downwards (DS) on age of oviposition and fed different diets varying in their protein to carbohydrate ratio. Error bars indicate the SE of the mean for wild flies, and the SE of the grand mean for replicate in CT and DS lines.

Body weight was affected by the selection regime, but also by the interaction between diet and sex (Table 1). Regardless of the selection regime, males fed the 0:1 P:C diet were lighter than the ones fed the 1:4 P:C diet (0:1–1:4: estimate = −0.64, *p* = 0.048), and no difference was detected between the other diets. In contrast, females fed the 0:1 P:C diet were lighter than the ones fed the 1:4 (0:1–1:4: estimate = −1.84, *p* < 0.001) and 1:2 (0:1–1:2: estimate = −2.53, *p* < 0.001) P:C diets (Figures 3A,B). In addition, females fed the intermediate diet were lighter than the ones fed with the best diet for daily egg production (1:4–1:2: estimate = −0.70, *p* = 0.028). CT flies were heavier than wild flies (CT—Wild: estimate = 1.19, *p* = 0.037), but no differences were found between other groups (Figures 3A,B).

## Discussion

We tested the hypothesis that the diet-mediated trade-off between lifespan and reproduction observed in female *C. cosyra* is associated with the balance between ROS production and antioxidant defences. Further, we predicted that variation in selection lines of *C. cosyra* differing in female reproductive scheduling and lifespan in both sexes would be associated with variation in oxidative damage and, or antioxidant protection. Accordingly, we tested the role of oxidative homeostasis in mediating life-history trade-offs observed at individual (i.e., physiological) and population (i.e., evolutionary) levels. Contrary to expectations, we found no difference in oxidative damage or total antioxidant capacity between lines subjected to different selection regimes. However, laboratory adapted lines (i.e., control and downward-selected lines) showed higher levels of lipid peroxidation and protein oxidation than wild flies. Interestingly, flies fed diets that supported female reproduction suffered greater oxidative damage to lipids. With regard to nutrient storage, flies selected for an earlier peak of fecundity (i.e., downward-selected flies) had lower lipid reserves. Protein content was lower in flies fed the diet promoting lifespan (i.e., 0:1 P:C), and lipid storage was also lower in flies from downward-selected lines fed this same diet.

If ROS mediate life-history trade-offs, we predicted that we would observe higher levels of oxidative damage accompanied by reduced or unchanged antioxidant protection in downward-selected lines. This is because our downward-selected lines are characterised by higher investment in early fecundity and shorter lives in comparison with laboratory adapted control lines. Although downward-selected flies did suffer higher oxidative damage than wild flies, they did not differ from control laboratory lines in their total antioxidant protection and even had lower oxidative damage when fed the diet promoting lifespan. These results contrast with a previous study on *C. cosyra* where the control lines were compared to upward-selected ones. In Malod et al. (2020), control lines with longer lives and higher fecundity had greater levels of lipid peroxidation and higher total antioxidant capacity. However, protein and carbohydrate sources were provided separately in Malod et al. (2020), which would allow flies to self-regulate towards their preferred protein to carbohydrate ratio. In contrast, flies in the present study had no choice but to feed on the P:C ratio of the diet they were assigned to. This variation in dietary manipulation may explain differences in results between studies. Taken together, this suggests that the apparent reduction in lifespan in downward-selected lines coupled with greater investment in reproduction is probably not driven by oxidative stress.

The potential role of ROS as a mediator of the trade-off between lifespan and reproduction was partially supported when nutrition was added to the balance. Either ROS production or repair of oxidative damage was probably greater in flies fed the diets that support reproduction (1:4 and 1:2 P:C). This is because lipid peroxidation was higher in flies fed these diets compared with flies provided with the diet promoting lifespan, but antioxidant protection did not change. We cannot exclude the hypothesis that those flies might have differed for other types of damage caused by oxidative stress (e.g., DNA damage) or antioxidant protection (e.g., antioxidant enzymes) that were not measured in this study. Even so, our results show that diets enhancing fecundity result in more oxidative damage to lipids, which may indicate a cost of reproduction (Metcalfe and Alonso-Alvarez, 2010). However, because lipid peroxidation levels did not increase as the P:C ratio increased, it is difficult to attribute the deleterious effects of the diets on lifespan to either a cost of reproduction or simply toxic effects of consuming excess proteins. Similar patterns have been observed in another tephritid fly species. In *B. dorsalis*, Chen et al. (2013) reported that the lowest TAC was found at 0:1 P:C ratio, the highest at 1:5 P:C ratio, and no difference found between two other intermediate ratios (1:100 and 1:30 P:C). However, when lipid peroxidation was measured, oxidative damage decreased with higher yeast to sugar ratios (Chen et al., 2013). The different patterns observed for oxidative damage between the current study and Chen et al. (2013) could originate from different experimental designs (e.g., lower P:C ratios used, different protein source: yeast vs. amino acid blends).

Male reproductive effort is complicated to measure in fruit flies and was not measured in the current study, it is therefore unclear how the diets affected male reproductive performance. Nevertheless, because oxidative damage and antioxidant protection patterns across diets did not differ between sexes, it may be that one of the male reproductive traits has perhaps been promoted by this diet. For example, in *Bactrocera tryoni* male reproductive success is decreased (lower mating propensity and shorter copulation duration) when males do not have access to hydrolysed yeast in their diet (Prabhu et al., 2008). A consequence of a similar response in *C. cosyra* could be the higher lipid peroxidation and total antioxidant capacity seen in males than in females, which might indicate a cost of reproduction in males. This has been shown in *Teleogryllus commodus*, where a diet related increase in male reproductive effort was associated with increased protein oxidation (Archer et al., 2015).

As individuals grow, it is anticipated that they store more nutrients, and this can be observed in tephritid flies, including *C. cosyra* (Weldon et al., 2016). In *C. cosyra*, there is sexual dimorphism in terms of body weight, with females being heavier. In line with this, heavier flies had higher soluble protein content, but lipid storage was not affected by body weight. The selection regime did not have much of an impact on the overall body nutrient composition. Only lipid reserves were affected. As predicted, lipid storage was lower in downward-selected flies than in the other groups. Nevertheless, this is an important observation as lipid reserves have been shown to decrease with age in fruit flies (Jacome et al., 1995; Warburg and Yuval, 1996). Although the experimental flies were of the same age, it may be that flies from the downward-selected group that have been selected for early reproductive effort deplete their lipid reserves at a faster rate than the other flies. This may be a result of downward-selected flies investing earlier in reproductive effort, as lipids are a primary source of energy but also essential components of oocytes and are stored in the fat body of the female (Arrese and Soulages, 2010). Our results are in line with past observations on *D. melanogaster* lines selected for postponed senescence. In comparison with control lines, postponed senescence selected flies with longer lifespan had higher lipid content (Service, 1987). Other studies suggest that low body fat is associated with long lifespan in lines selected for postponed senescence in *D. melanogaster* and that an increase in body fat leads to shorter lifespan (Moghadam et al., 2015). However, lines selected for postponed senescence used by Moghadam et al. (2015) had a higher early fecundity than control flies, whereas it was the opposite in lines used by Service (1987). Hence, the difference in the effect of lipid storage on lifespan might be due to differences coming from the selection regime. In terms of early reproductive effort, in the current study, flies from the downward-selected lines have been shown to outperform control flies or upward-selected flies (Malod et al., 2021). Therefore, the effects of the selection regime are similar to those observed by Service (1987). Furthermore, using an experimental selection approach or not, other studies have shown that long lifespan is associated with higher lipid storage (Lee et al., 2008; Hansen et al., 2013).

In conclusion, this study suggests that oxidative stress balance alone may not act as a mediator of the trade-off between lifespan and reproduction observed in different populations of female *C. cosyra*. However, at the individual level our results indicate that oxidative damage may be a cost of elevated female reproductive investment. This is because we observed higher levels of lipid peroxidation in flies fed diets that benefit fecundity at the expense of lifespan, indicating by extension more elevated ROS production as a cost of reproduction. Moreover, our results provided mixed support to the idea that lipid reserves benefit lifespan. On the one hand, downward-selected lines characterised by shorter lifespan stored less lipids overall. However, the diet promoting lifespan was associated with lower lipid reserves in downward-selected lines. This may indicate that these lines that already have low lipid reserves and favour early reproduction over lifespan have depleted their lipid stores. Further studies should investigate antioxidant enzyme activity, such as superoxide dismutase or catalase, to contribute to a better understanding of the effect of reproductive scheduling on oxidative stress. In addition, a larger range of P:C ratios should also be investigated to ascertain whether flies fed diets containing proteins have higher levels of oxidative damage due to the cost of reproduction or because of potential toxic effects of proteins. Finally, this study shows that the factors that regulate trade-offs may interact together in a complex manner and may depend on the evolutionary history of the tested populations.

## Data Availability Statement

Data for this study are openly available in figshare at: http://doi.org/10.25403/UPresearchdata.19107266, reference number 19107266.

## Author Contributions

CA, SN, and CW conceived and designed the study. ER and KM conducted the experiments and collected the data. KM performed the statistical analyses. KM, CA, and CW wrote the manuscript. All authors contributed to the article and approved the submitted version.

## Funding

KM was supported by a National Research Foundation Competitive Programme for Rated Researchers awarded to CW, SN, and CA (no: 93686).

## Conflict of Interest

The authors declare that the research was conducted in the absence of any commercial or financial relationships that could be construed as a potential conflict of interest.

## Publisher’s Note

All claims expressed in this article are solely those of the authors and do not necessarily represent those of their affiliated organizations, or those of the publisher, the editors and the reviewers. Any product that may be evaluated in this article, or claim that may be made by its manufacturer, is not guaranteed or endorsed by the publisher.
