# Supplementary material for: Oxidative Damage Is Influenced by Diet But Unaffected by Selection for Early Age of Oviposition in the Marula Fly, Ceratitis cosyra (Diptera: Tephritidae)
Source: Front Physiol. 2022 Feb 28;13:794979. doi: 10.3389/fphys.2022.794979 (PMC8918681; doi:10.3389/fphys.2022.794979)
Supplement: Supplementary file 1 [file Data_Sheet_1.docx]

**Oxidative damage is influenced by diet but unaffected by selection for early age of oviposition in the marula fly, *Ceratitis cosyra* (Diptera: Tephritidae)**

**Kevin Malod^a†^, Esther E. du Rand^a^, C. Ruth Archer^b^, Susan W. Nicolson^a^, Christopher W. Weldon^a*^**

*^a^Department of Zoology and Entomology, University of Pretoria, Private Bag X20, Hatfield 0028, South Africa.*

*^b^Institute for Evolutionary Ecology and Conservation Genomics, University of Ulm, Ulm, Germany.*

^†^*Current address: Department of Conservation Ecology and Entomology, Faculty of AgriSciences, Stellenboch University, Stellenbosch, South Africa*

**Corresponding author: E-mail:* [*cwweldon@zoology.up.ac.za*](mailto:cwweldon@zoology.up.ac.za)


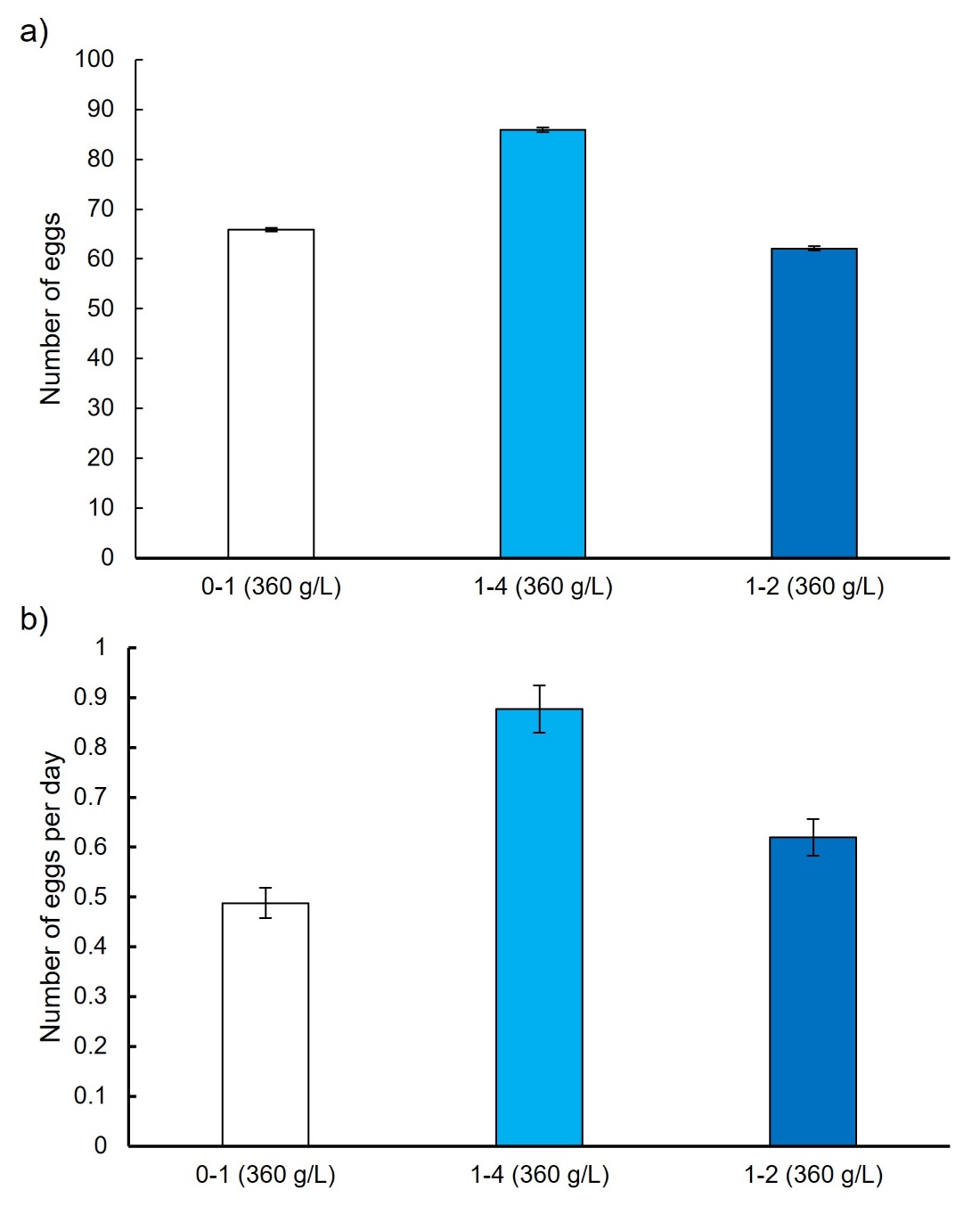


**Figure S1. Average lifetime egg production (a) and daily egg production (b) in control females fed one of following diets that vary in their protein to carbohydrate (P:C) ratio: 0:1 P:C, 1:4 P:C and 1:2 P:C. Error bars represent the standard error to the mean.**

Table S1. Experimental diets fed to female and male *C. cosyra* in either no-choice or choice experiment.

| P:C | Concentration (g/L) | Sucrose (g/L) | Amino Acids (g/L) | Vitamin (g/L) | Cholesterol (g/L) | RNA (g/L) | Wesson salt (g/L) |
| --- | --- | --- | --- | --- | --- | --- | --- |
| 0:1 | 360 | 360.00 | 0.00 | 3.6 | 4.0 | 10.0 | 10.0 |
| 1:4 | 360 | 288.00 | 72.00 | 3.6 | 4.0 | 10.0 | 10.0 |
| 1:2 | 360 | 240.00 | 120.00 | 3.6 | 4.0 | 10.0 | 10.0 |
